# No Clear Effect of Initiating Vaccination against Common Endemic Infections on the Amounts of Prescribed Antimicrobials for Danish Weaner and Finishing Pigs during 2007–2013

**DOI:** 10.3389/fvets.2016.00120

**Published:** 2017-01-16

**Authors:** Amanda Brinch Kruse, Leonardo Víctor de Knegt, Liza Rosenbaum Nielsen, Lis Alban

**Affiliations:** ^1^Department of Large Animal Sciences, Faculty of Health and Medical Sciences, University of Copenhagen, Frederiksberg, Denmark; ^2^Danish Agriculture & Food Council, Aarhus, Denmark

**Keywords:** antimicrobial, vaccination, pig production, *Mycoplasma hyopneumoniae*, porcine circovirus type II, *Actinobacillus pleuropneumoniae*, porcine reproductive and respiratory syndrome virus, *Lawsonia intracellularis*

## Abstract

It is often stated that vaccines may help reduce antimicrobial use in swine production. However, limited evidence is available outside clinical trials. We studied the change in amounts of antimicrobials prescribed for weaners and finishers in herds following initiation of vaccination against five common endemic infections: *Mycoplasma hyopneumoniae, Actinobacillus pleuropneumoniae*, porcine circovirus type II, porcine reproductive and respiratory syndrome virus, and *Lawsonia intracellularis*. Comparison was made to the change after a randomly selected date in herds not vaccinating against each of the infections. Danish sow herds initiating vaccination during 2007–2013 were included (69–334 herds, depending on the analysis). Danish sow herds with no use of the vaccine in question were included as non-exposed herds (130–570 herds, depending on the analysis). Antimicrobial prescriptions for weaners in sow herds and for finishers in receiving herds were extracted from the VetStat database for a period of 12 months before and 6–18 months after the first purchase of vaccine, or random date and quantified as average animal daily doses (ADDs) per 100 animals per day. The herd-level difference between ADD in the period after and before vaccination was the outcome in linear regression models for weaner pigs, and linear mixed-effects models for finishing pigs, taking into account sow herds delivering pigs to two or more finisher herds. Three plausible risk factors (Baseline ADD, purchase of specific vaccine, purchase of other vaccines) and five confounders (herd size, export and herd health status, year and season) were initially considered in all 10 models. The main significant effect in all models was the Baseline ADD; the higher the Baseline ADD was for weaner and finishing pigs, the larger the decrease in ADD was following vaccination (or random date for non-vaccinating herds). Regardless of vaccination status, almost equal proportions of herds experienced a decrease and an increase in ADD resulting in no overall Change in ADD. Furthermore, only minor effects were found, when vaccinations were used in combination. In conclusion, this study provided little support for the hypothesis that vaccination against five common endemic diseases provides a plausible general strategy to reduce antimicrobial use in Danish pig herds.

## Introduction

Due to its large production, the Danish pig sector accounts for 76% of the total amount of antimicrobial substances used for livestock production per year in the country (1). Official focus on reducing antimicrobial use has, therefore, been on the pig production. The Danish Government and the swine industry have put in place several initiatives to try to mitigate the potential risk related to the development of antimicrobial-resistant bacteria. One of these initiatives, “the Yellow Card Scheme,” which identifies and warns livestock farmers using above a given permitted limit of antimicrobials, was introduced in 2010 and is managed by the Danish Veterinary Authorities. The antimicrobial use decreased after the introduction of the Yellow Card Scheme (2). From 2010 to 2014, there was a 14% reduction in the antimicrobial treatment proportion, measured as defined animal daily doses (ADDs) per 1,000 animals per day across the total Danish pig production (1). The pig industry’s goal is a further reduction by 10% before 2020 (3). To achieve this, relevant and effective strategies that can minimize the need for treatment with antimicrobials in the pig production are needed.

For animal welfare and productivity reasons, diseased animals should be treated. However, an increased or improved use of vaccination has been suggested as a potential strategy to prevent specific diseases and/or secondary infections (4). Today, the majority of Danish sows are being vaccinated against several pathogens as a standard procedure. On the other hand, vaccination of offspring is not used to the same extent in Denmark as in other EU countries with a similar pig production. There may be different explanations for this—one is the extended use of a controlled program for specific pathogen-free (SPF) production of piglets. However, the use of several vaccines has been on the increase lately, especially since “the Yellow Card Scheme” was adopted by the Danish Veterinary Authorities (2).

In Danish pig production, the majority of antimicrobials are used for treatment of gastrointestinal and respiratory indications in weaner pigs from 7–30 kg, followed by treatment of gastrointestinal indications in finishing pigs (VetStat data, unpublished). *Mycoplasma hyopneumoniae* (MYC), porcine circovirus type II (PCV2), *Actinobacillus pleuropneumoniae* (APP), porcine reproductive and respiratory syndrome (PRRS) virus, and *Lawsonia intracellularis* (LAW) represent some of the most important disease agents related to these indications, which are also preventable through vaccination of breeding animals and/or offspring.

*Mycoplasma hyopneumoniae* is a bacterium causing enzootic pneumonia in pigs. Enzootic pneumonia is most often seen in finishing pigs, where it is associated with productivity losses. The bacteria are considered to be present in all Danish conventional pig herds and in 67% of SPF herds (5). MYC in itself does not necessarily cause disease problems in infected herds. However, associated secondary infections may aggravate clinical signs, and increase the need for treatment (6). Vaccination against MYC would, therefore, be expected to reduce the need for antimicrobial treatment. The effect of vaccination against MYC has previously been shown to have a positive effect on growth and reduced lung lesions (7–9). There are several vaccines against MYC available on the Danish market. MYC corresponded to 36% of the vaccine dosages prescribed in 2013, being, therefore, the most frequently used type of vaccine in pig production (10).

Porcine circovirus type II is a virus associated with several different clinical signs in pigs. The virus is considered present in nearly all Danish pig herds, without necessarily causing disease problems. Previously, the main disease problem related to PCV2 in Danish pig production was postweaning multisystemic wasting syndrome (PMWS) in weaner pigs. Nowadays, problems are mainly related to reduced growth and increased mortality in finishing pigs. PCV2 has an immuno-suppressive effect, which potentiates the impact of other pathogens, resulting in a need for antimicrobial treatment (11). Therefore, vaccination against PCV2 could potentially reduce disease occurrence and, consequently, the use of antimicrobials. In fact, after the adoption of the Yellow Card Scheme, a 31% increase in the use of PCV2-vaccines was seen over 1 year in Danish pig production (2). Vaccination against PCV2 has been shown to result in increased growth rate and reduced mortality in finishers (12), as well as reduced antimicrobial use (13, 14). Vaccines against PCV2 are the second most frequently used group of vaccines in Danish pig production, representing 26% of the vaccine dosages prescribed in 2013 (10).

*Actinobacillus pleuropneumoniae* is a bacterium causing pleuropneumonia in pigs and is associated with reduced growth and increased mortality, primarily in finishing pigs. There are 15 different serotypes producing a combination of two or more of the four toxins responsible for the pathology leading to disease in pigs (15). The most prevalent serotypes in Denmark are serotypes 2, 5, 6, 5, and 7. Most SPF herds are free from APP. However, serotype 6 is present in 26% of SPF herds, serotype 2 in 17%, serotype 7 in 0.4%, and serotype 5 in 0.1% (5). Studies have shown that vaccines against APP can reduce the prevalence of pleuritis (16, 17). Vaccines against APP are the third most frequently used type of vaccines in Danish pig production, representing 8% of the vaccine dosages prescribed in 2013 (10). It could be expected that preventing APP by using vaccination would reduce the treatment of this bacterial infection.

Porcine reproductive and respiratory syndrome virus multiplies in macrophages in the lungs, thereby making pigs more susceptible to bacterial infections, such as infections with *Streptococcus suis* (18). There are two different strains; the United States (US) strain and the European strain, which are both present in the Danish national pig herd. Among SPF herds, 27% are infected with the US strain, whereas 20% are infected with the European strain. The two strains are causing similar clinical signs in pigs, mainly reproductive failure and respiratory distress (5). Vaccines against PRRS virus represent 2% of the vaccines dosages prescribed (10). The use of vaccines against PRRS in Denmark has been on a more or less constant lower level, compared to the use of vaccines against MYC, PCV2, and APP (VetStat data, unpublished). There are currently two modified-live vaccines and two inactivated vaccines against PRRS on the Danish market (19).

*Lawsonia intracellularis* is one of the predominant agents responsible for the development of porcine proliferative enteropathy, resulting in diarrhea in weaner and finishing pigs (20). The herd-level prevalence of LAW in Danish herds is above 90% (21), but the infection does not necessarily lead to clinical disease. Still, substantial amounts of antimicrobials are used for the treatment of gastrointestinal indications in Danish pigs. Therefore, prevention of diarrhea caused by LAW is likely to lead to reduced use of antimicrobials. There is currently only one vaccine available for prevention of LAW, and it accounted for 3% of the vaccine dosages prescribed for pigs in 2013 (10). This implies that it is used to nearly the same extent as vaccines against PRRS, and the purchase of this vaccine has increased since 2010 (VetStat data, unpublished). Previous studies have found reduced mortality and increased growth followed by vaccination against LAW (22), as well as reduced antimicrobial use (23).

Initiating vaccination in a herd represents an extra production cost. Hence, it is important for the farmer to know the cost-efficiency of vaccines in different situations. Previous studies testing the effect of vaccines against MYC, PCV2, APP, PRRS, or LAW have mainly investigated the effect on production or health parameters. Few studies have investigated the effect of vaccination on antimicrobial usage, and usually, the effect has only been assessed in one farm at a time, not allowing for any generalization of results. A recent study by Temtem et al. (10) evaluated the effect of vaccines against MYC, PCV2, and LAW on antimicrobial use in 1,513 Danish pig farms, using a cross-sectional study design to compare the total amount of antimicrobials prescribed in 2013 in herds with and without vaccination against one or more of MYC, PCV2, and LAW. However, the date of initiation of vaccination and other possible important risk factors and confounders were not taken into account.

The objective of the present study was to estimate the effect of initiating vaccination against MYC, PCV2, APP, PRRS, and LAW on the change in antimicrobial prescription in weaner and finishing pigs at herd-level, while taking into account plausible associated risk factors and confounders. To evaluate whether eventual detected effects were actually related to vaccination rather than general trends in the target population, the change in antimicrobial prescription in vaccinating herds was compared to the change in antimicrobial prescription in randomly selected comparable periods for herds not vaccinating against the specific vaccine in question.

## Materials and Methods

### Herd Enrollment

**Figure 1:**
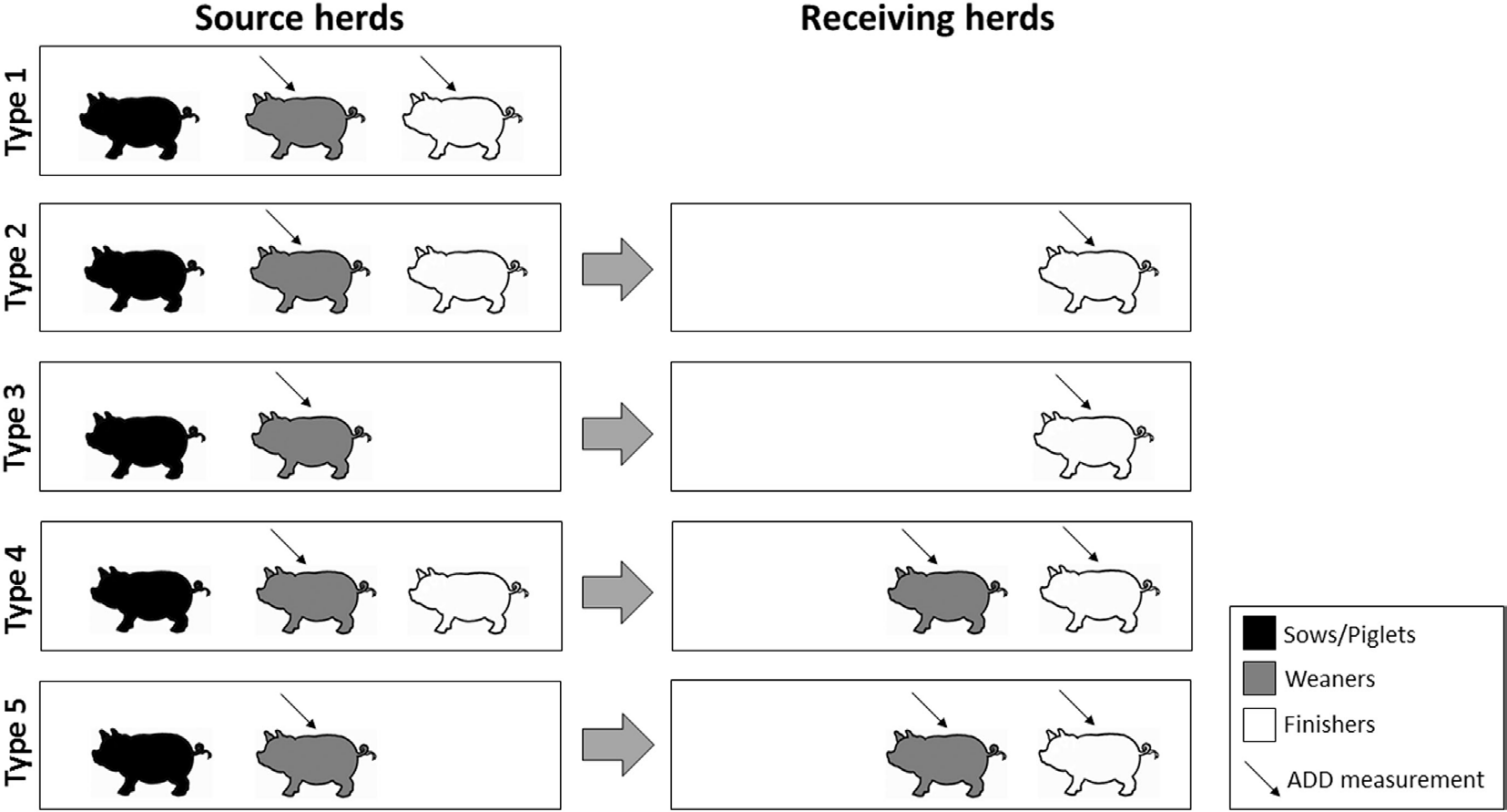
**Illustration of the types of source herds included in the study, characterized by herd composition and types of pigs moved to other herds**.

Full-line conventional pig herds were identified using yearly data extractions from the Central Husbandry Register (CHR) and quarterly extractions of movements between pig herds from the Danish Pig Movement Database. The following types of herds were identified and included (Figure 1):

- Type 1: farrow-to-finisher herds, which contained age groups *Sows, Weaners*, and *Finishers* registered under one CHR number (implying one geographical location). In order to be considered a farrow-to-finisher production, the number of Weaner and Finisher pen places had to be at least 1.5 times the number of sow pen places, indicating that at least part of the offspring remained in the herd until the finishing stage.
- Type 2: herds with age groups *Sows, Weaners*, and *Finishers* registered under one CHR number (source herd) and with movement of growing pigs to herds with age group *Finishers* (receiving herd).
- Type 3: herds with age groups *Sows* and *Weaners* registered under one CHR number (source herd) and with movement of growing pigs to herds with age group *Finishers* (receiving herd).
- Type 4: herds with age groups *Sows, Weaners*, and *Finishers* registered under one CHR number (source herd) and with movement of growing pigs to herds with age groups *Weaners* and *Finishers* (receiving herd).
- Type 5: herds with age groups *Sows* and *Weaners* registered under one CHR number (source herd) and with movement of growing pigs to herds with age groups *Weaners* and *Finishers* (receiving herd).

It was a requirement that each receiving herd only received pigs from one source herd in each quarter of a year, whereas source herds could deliver pigs to more than one finisher pig herd. Moreover, only source herds with a minimum of 100 sow pen places and a minimum number of weaner pen places equal to or higher than 1.5 times the number of sow pen places were included, to make sure these herds were not sow-only or weaner-only. For receiving herds to be included, a minimum of 100 finisher pen places was required.

### Purchase of Vaccination

To study the effect of initiating vaccination against MYC, PCV2, APP, PRRS, and LAW, 10 different longitudinal studies were carried out on historical data; for each vaccine group, one study was made on the effect on antimicrobial use in weaner and finishing pigs. Purchase of vaccines for Danish pig herds is recorded in the Danish VetStat Database (VetStat). Data used in this study were raw historical data from VetStat, retrieved on June 1, 2015. Purchase of all vaccine products against the five disease agents was extracted for the source herds. All herds with their first purchase of a vaccine under study between January 1, 2007, and December 31, 2013, were included. A sow herd was considered to have initiated vaccination in this period and was included in the analyses if it fulfilled the following criteria:

1. No prior purchase of the vaccine in question either in the source herd or receiving herds from January 1, 2005.
2. Purchase of the vaccine for at least 1 year following the first purchase.
3. Purchase of more than a minimum threshold of vaccine doses per year per sow. The minimum of doses was determined based on evaluation of a histogram showing the distribution of vaccine coverage. The vaccine coverage was calculated as the number of doses in the first year of vaccination divided by the number of sows registered in the individual herd multiplied with 25, representing the average number of weaned pigs per sow per year in Denmark. For MYC and PCV2, the threshold was set at >0.5 and <1.5 corresponding to herds with a vaccine coverage between 50 and 150%. This large a margin around the 100% coverage was needed in order to include those herds representing the area on the histogram with the majority of the observations. For LAW, APP, and PRRS, the threshold was lowered in order to avoid too many herds being excluded due to this criterion. Therefore, only an upper threshold was set at <1.5.

Non-vaccinating herds were identified based on extraction of all active source herds in CHR with no purchase of the vaccine in question, at any time within the period between January 1, 2005, and April 30, 2015. A herd was considered active when having recordings of antimicrobial prescriptions in VetStat for all quarters, in a period of 12 months before and 18 months after the first vaccine purchase.

For each analysis, information about purchase of the remaining four types of vaccines within the period studied for each herd was included. For this, data on the general purchase of the vaccines in the period from January 1, 2005, until April 30, 2015, were extracted and summarized by quarter of the year, to be able to match correctly with the selected study period for each herd included. For a herd to be considered using other vaccines, the herd had to have purchased the given vaccine for at least 1 year in total and within at least one quarter of the study period for the individual herd.

### Antimicrobial Use

Herd-level antimicrobial prescription data extracted from the raw VetStat data were used in this study as a proxy for antimicrobial use. All prescriptions of antimicrobials, irrespective of indication for weaner and finishing pigs, were included in the study. For farrow-to-finisher herds, data on antimicrobial prescription for weaner and finishing pigs were extracted for the individual herd. For full-line herds identified using movement data, data on antimicrobial prescription for weaner pigs were extracted from the source herd, while for finishing pigs data were extracted from the receiving herds (Figure 1).

Antimicrobial data for weaner and finishing pigs were extracted for a period of 2.5 years. This consisted of data from 1 year before vaccination was initiated, until 1.5 years after vaccination was initiated. The period of the first 6 months after vaccination was initiated was considered a transition period, in which not all weaner and finishing pigs entering the stables had been vaccinated yet. Data from this period were excluded for the data analyses. Each prescription was converted into a number of ADD, using standardized doses per antimicrobial product developed by the Danish Veterinary and Food Administration. The ADD takes into account a standard average weight of a weaner pig (15 kg) and a finishing pig (50 kg), as well as the total amount and dose of the antimicrobial prescribed. All prescriptions in ADD were divided into quarterly prescriptions for each herd, over the given period of 2.5 years selected for analysis. Each herd had to have prescriptions in each of the quarters within this study period, in order to be considered an active producing herd and, therefore, be included in the study. The number of weaner and finishing pigs in each herd at the time of vaccination was provided by data from the CHR. These numbers were used in the calculation of the average ADD per 100 weaners per day, and of ADD per 100 finishers per day, in the 1-year period before (Baseline ADD) and 6–18 months after vaccination was initiated (ADD After) in each herd. The change in amount of prescribed antimicrobials (Change in ADD) following initiation of vaccination was calculated for each herd by subtracting Baseline ADD from the ADD After.

For herds not vaccinating against a specific pathogen (e.g., APP, MYC, etc.), a random date was chosen in the same period as for the vaccinating herds (between January 1, 2007, and December 31, 2013), and the same set-up was used around this date; antimicrobial prescription during 1 year before the random date was included, followed by 6 months of data, which were excluded, and then data on antimicrobial prescription covering 1 year were included. Also, Change in ADD was calculated for these herds, which acted as a comparison group. It should be noted that, in the analyses, it was still taken into account whether these herds that were not exposed to the specific vaccine in question (e.g., MYC) were vaccinated against any of the other vaccines (i.e., PCV2, APP, PRRS, and/or LAW).

On rare occasions, prescription entry errors occur in the VetStat database, resulting in either negative or extreme values, when calculating ADD over a selected time period. When negative values of ADD were identified, the corresponding herd was excluded from the study. Also, a few herds with extremely high ADD values (>60 ADD/100 weaners/day and >20 ADD/100 finishers/day) were excluded, as these most likely reflected recording errors or dramatic unregistered changes in the herd population.

### Description of Variables

In all models, the outcome variable was the Change in ADD, and this variable was included as a continuous variable after checking for linearity. Three variables were tested as potential explanatory variables for Change in ADD:

- *Vaccination*: categorical variable with two levels; “Yes” and “No,” representing vaccinating (exposed) and non-vaccinating (non-exposed) herds, respectively.
- *Baseline ADD*: continuous variable, representing a baseline measure of antimicrobial use. It was estimated as the antimicrobial prescription 1 year before vaccination (or random date for the non-vaccinating group), in average ADD per 100 animals per day, for weaned and finishing pig.
- *Other vaccines*: four categorical variables, one for each of the other four vaccines than the one under investigation in each study, with two levels; “Yes” and “No,” representing source herds that were or not using another vaccine when vaccination with the study-specific vaccine started.

In addition, five variables were included as potential confounders:

- *Sows*: the number of sows in the individual source herd was included as a continuous variable, representing the herd size.
- *Year*: the year of the first purchase of vaccine (or random date) was included as a categorical variable, representing the different changes and levels of antimicrobial use, which has been seen in Danish pig production between 2007 and 2013. After initial analyses with individual years, this variable was further grouped into “Before 2010” (<2010) and “After 2010” (≥2010), representing the period before and after the Yellow Card Scheme was implemented in Denmark.
- *Season*: because it is known that antimicrobial use can fluctuate by season and a farmer may be more likely to initiate vaccination during seasons with high antimicrobial use, the season might confound the estimate of the effect of initiating vaccination. Therefore, the quarter of the year of the first purchase of vaccine (or random date for non-vaccinating herds) was included, to account for season as a categorical variable with four levels; “1,” “2,” “3,” and “4,” representing the first to the fourth quarter of the year.
- *SPF*: categorical variable with two levels; “SPF” and “Non-SPF,” representing source herds enrolled or not in the SPF system at the time of vaccination (or random date), respectively. Information about enrollment of each herd in the Danish SPF system was provided by SEGES Pig Research Centre (SEGES). The SPF variable was used as a measure of herd health status and considered as a potential confounder, as herds in the SPF system might have a better health status than non-SPF herds, which may influence the antimicrobial use in those herds.
- *Export*: categorical variable with two levels; “Yes” and “No,” representing source herds with and without export of growing pigs, respectively. For each herd, information about exporting of animals was assessed using the Danish Pig Movement Database, which contains all movements of animals from Danish herds to other countries. Besides having moved animals from the source to the receiving herd, some of the herds had also exported either 7 or 30 kg weaner pigs to other countries. This information was included as a potential confounder in the analyses, as importers might demand that pigs are vaccinated against specific diseases, even if the Danish herd was not infected with the given disease agent.

Continuous variables were plotted against the outcome variable and against each other, to visually check for linearity and correlations. For categorical variables, distributions were checked for a reasonable number of observations in each variable level.

### Statistical Analyses

All statistical analyses and data management were carried out using the software R version 3.1.3. Separate data analysis was conducted for each of the 10 models, representing the effect of the five types of vaccines in weaner and finishing pig herds. General linear regression models were used to model the Change in ADD for weaner pigs as a function of the potential explanatory variables and covariates.

The same variables were used to model the Change in ADD for finishers in a linear mixed-effects model, using the *lme4* package in R (24). Source herd was included as a random effect to account for clustering of antimicrobial use patterns in herds with animals originating from the same source herd, and all other explanatory factors and covariates were included as fixed effects. First, univariable models between each explanatory variable and the outcome were assessed, and if associations showed *P* < 0.20, the variable was included in the multivariable model. However, to prevent misinterpretation in the face of poor data availability, if one of the stratified groups in a categorical variable contained fewer than five observations, the variable was not included in the multivariable model. The final multivariable model was identified by backwards-stepwise elimination of non-significant predictors, using the drop1-function in R. Significant two-way interactions of all main effects were checked one by one. The criterion for keeping a predictor or a two-way interaction in the model was *P* < 0.05, and models were compared using Akaike’s Information Criteria (AIC), with the AIC closest to 0 indicating the best model. Confounding was assessed by evaluating the models with and without each of the potential confounders. A variable was considered a confounder, if it changed the parameter estimates of any of the other significant variables by >20%. When an interaction between vaccination with the study-specific vaccine and one of the other vaccines was statistically significant, the two were recoded as a four-way variable, to be included in the final multivariable model, allowing for the estimation of the effect and significance of each category defined by the pairwise combination of vaccination statuses (Yes/Yes, No/No, Yes/No, No/Yes). The statistical significance of differences observed between the four categories was assessed with Tukey’s “Honest Significant Difference” method, using the *multcomp* package in R. The final models were presented with parameter estimates including SE and *P*-values, as well as the explanatory degree of the model.

The distribution of residuals was checked for normality using residual plots. The explanatory degree of the general linear regression models was assessed using the *R*^2^. ‘The explanatory degree’ of the mixed-effects models was assessed using the marginal pseudo-*R*^2^: (*R*_e_ − *R*_fm_)/*R*_e_, where *R*_e_ is the residual variance of the model, only containing the random effect of source herd, and *R*_fm_ is the residual variance of the final model (25).

## Results

### Descriptive Statistics

There were small, characteristic differences between vaccinating and non-vaccinating herds, when looking at the descriptive statistics; for the majority of the studies, the Change in ADD was lowest and the Baseline highest in the vaccinating group. Also, the vaccinating groups consisted of larger herds, represented by the number of sows in the source herd, when compared to non-vaccinating herds (Tables S1–S10 in Supplementary Material).

There were no substantial differences between the mean and median values for Change in ADD, Baseline ADD, and Sows. Therefore, it was chosen to present the mean. Overall, the mean Change in ADD was close to 0, but with a large range, especially for weaner pigs. As expected, the largest Baseline ADD was found among weaner pigs, with a nearly five times higher Baseline ADD than observed for finishers. The mean number of sows in the farrow-to-finisher and source herds only differed 1–7% between the studies on effect in weaner and finishing (Table S11 in Supplementary Material). This happened because nearly the same source herds were used in the analyses for weaners and finishers for each type of vaccine.

For each of the 10 studies, there were between 71 and 334 vaccinating source herds delivering pigs to between 89 and 365 receiving herds. For the group of non-vaccinating herds, there were between 130 and 570 source herds delivering pigs to between 158 and 662 receiving herds, in each of the studies.

### Analytical Statistics

A summary of the main findings from each of the final regression models is presented in Table 1. Detailed results from the models can be found in Tables S12–S16 in Supplementary Material.

**Table 1:** **Summarized^a^ results of the final linear regression models (weaners) and linear mixed models with random effects of potential confounders (finishers) predicting the change in antimicrobial consumption after initiation of vaccination in selected Danish swine herds between 2007 and 2013**.

Vaccine	Age group	Statistically significant effects				*R*^2^
		Vaccine	Baseline	Other individual variables	Interactions	
*Mycoplasma hyopneumoniae*	Weaners	No	Yes	*Actinobacillus pleuropneumoniae* (APP) vaccine	Baseline × year	0.25
MYC	Finishers	No	Yes	Specific pathogen-free	No	0.27
Porcine circovirus type II (PCV2)	Weaners	No	Yes	Year Export	No	0.27
PCV2	Finishers	No	Yes	Year Number of sows	No	0.24
APP	Weaners	No	Yes	Number of sows	Number of sows × year Baseline × year	0.26
APP	Finishers	No	Yes	No	No	0.29
Porcine reproductive and respiratory syndrome (PRRS)	Weaners	No	Yes	Year APP vaccine	No	0.21
PRRS	Finishers	No	Yes	No	Baseline × year	0.24
*Lawsonia intracellularis*	Weaners	No	Yes	Number of sows	Baseline × year	0.21
*LAW*	Finishers	Yes^b^	Yes	No	Vaccine × PRRS vac.	0.30

*^a^The complete list of variables, coefficients, SE, and p-values for all models are shown in Tables S12–S16 in Supplementary Material*.

*^b^Non-significant as an isolated variable but significant as an interaction with another vaccine*.

**Figure 2:**
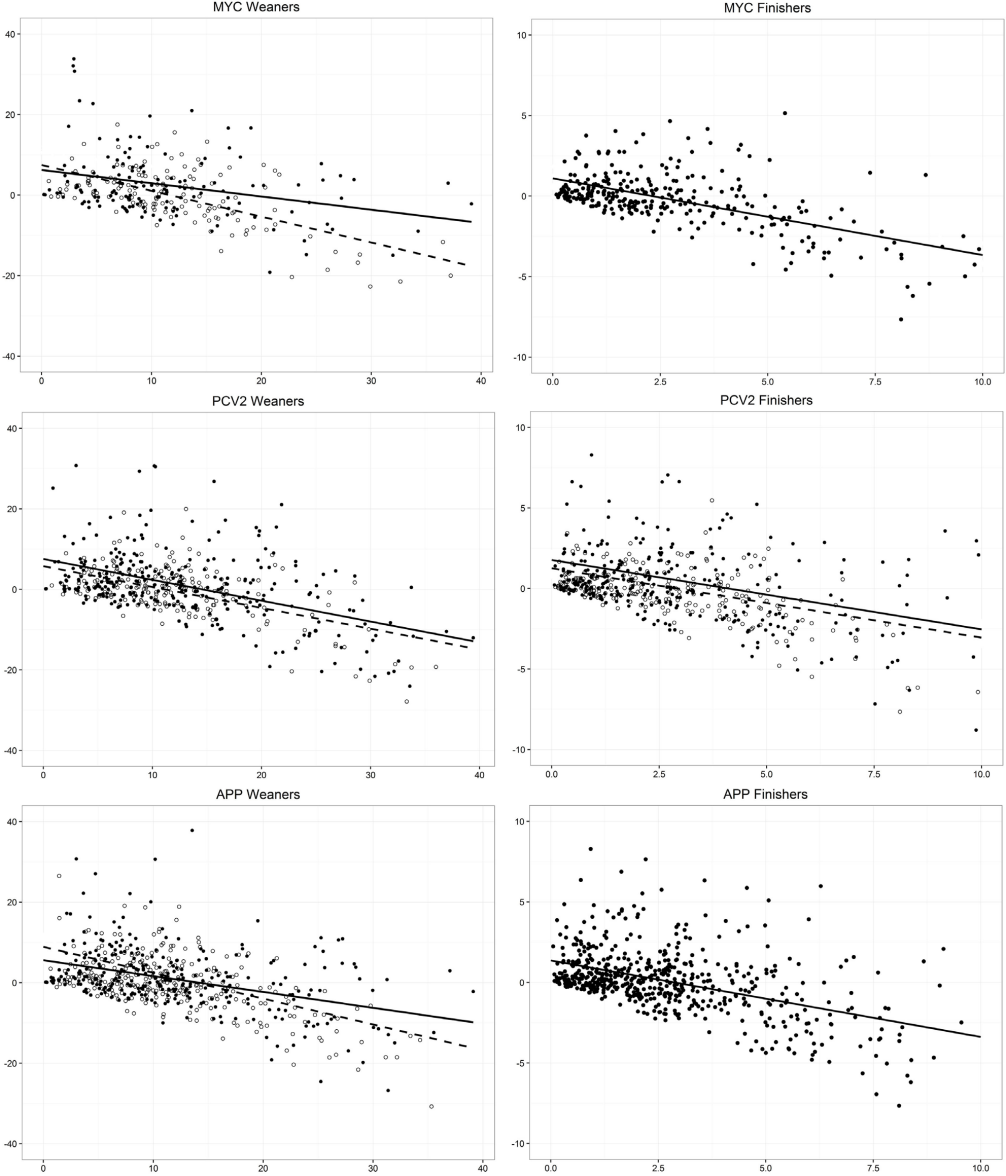

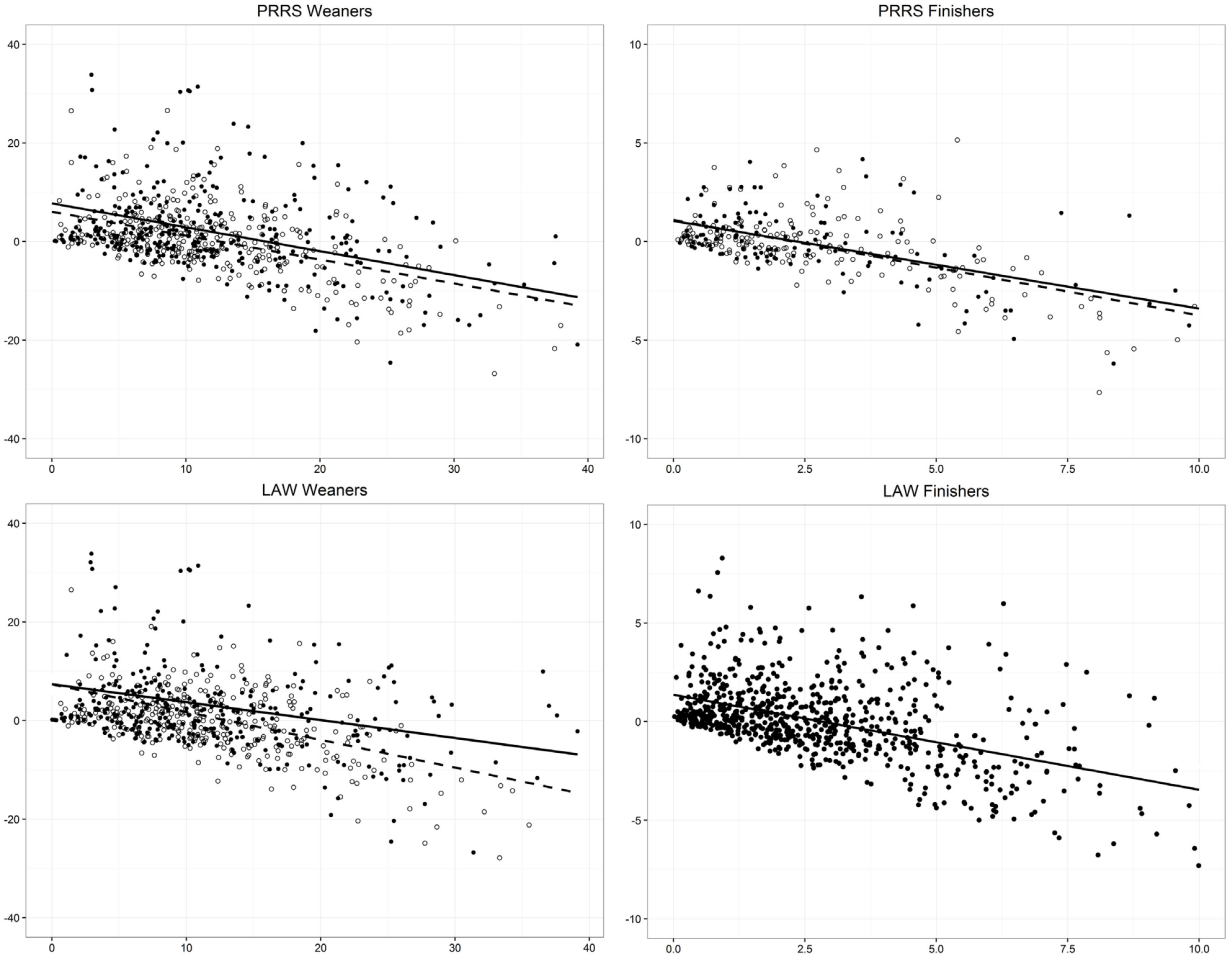
**Model-predicted associations between Baseline ADD (horizontal axis) and Change in ADD (vertical axis), before (continuous line, based on black dots) and after (dashed line, based on grey dots) 2010, in groups of Danish swine herds that initiated vaccination or not against the five different endemic agents under study in 2007–2013**. Each graph illustrates one model derived from vaccine- and production type-specific dataset. In models with only one (continuous) line, there was no significant effect of year, i.e. before vs. after 2010, in which the Yellow Card Scheme was initiated.

**Figure 3:**
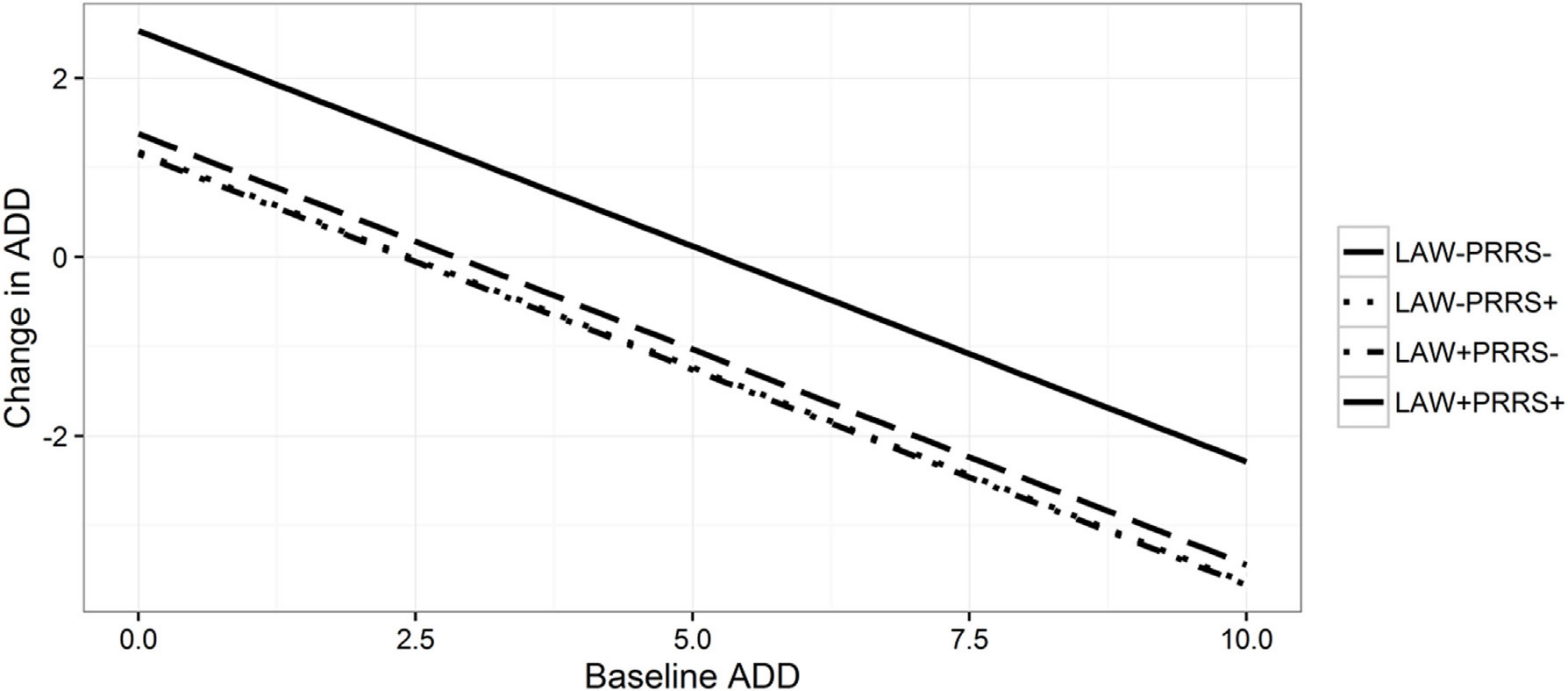
**Model-predicted associations between Baseline ADD per 100 finishers per day (horizontal axis) and Change in ADD (vertical axis) in groups of Danish swine herds that initiated *Lawsonia intracellularis* vaccination or not, while also vaccinating or not against porcine reproductive and respiratory syndrome in 2007–2013**.

No study-specific vaccinations were found to have a significant impact on the antimicrobial consumption, when analyzed independently. The baseline antimicrobial consumption before initiation of vaccination (Baseline ADD) was the only consistently significant independent variable in all models, indicating that herds with a higher Baseline ADD obtained a larger reduction in ADD, when compared to herds with a lower Baseline ADD. The interaction between Baseline ADD and vaccination status was non-significant in all models, meaning that the effect of Baseline ADD was the same for herds initiating vaccination as for non-vaccinating herds. For those reasons, plots presented in this manuscript are based on the effect of Baseline ADD on Change in ADD, added with specific variables of interest, depending on the case (Figures 2 and 3).

For weaners, all five final models showed an effect of year, either as a direct effect or as interacting effect with Baseline ADD, while for finishers this was only the case for two of the five models (Table 1). The effect of year in all seven models showed that a larger decrease in ADD was seen after 2010, when compared to the period before 2010. The effect was the same, irrespective of vaccination status, since no interaction between year and vaccination was found in any of the models. The effect of Baseline ADD on Change in ADD according to year (when significant) is shown for all models in Figure 2.

The effect of initiating study-specific vaccinations was only significant in an interaction with the use of another vaccine in the same period for LAW and PRRS vaccination in finishers (Table 1). An increase in ADD was observed for herds using both LAW and PRRS, when compared to non-vaccinating herds (Figure 3; Table S16 in Supplementary Material). In addition, the use of APP vaccines had a positive effect on the Change in ADD for weaners in the PRRS-model, implying that a larger increase in ADD is seen in herds with use of APP vaccines. For the latter, there were no significant interaction between initiating vaccination against PRRS and using APP vaccines, which is why this effect was general for both vaccinating and non-vaccinating herds (Table 1; Table S15 in Supplementary Material).

The number of sows, representing herd size, was significantly associated with an increase in ADD in the PCV2 model for finishers, as well as in the LAW and APP models for weaners. For the latter model, the number of sows was only associated with an increase in ADD in the period before 2010, which was shown by the significant interaction between year and Sows (Table 1).

As for variables Export and SPF, the model estimates revealed that exporting herds had a larger increase in ADD for weaners than non-exporting herds in the PCV2-model (Table 1; Table S13 in Supplementary Material). In the MYC model, SPF herds had a larger increase in ADD for finishers, when compared to Non-SPF herds (Table S12 in Supplementary Material). Again, these effects were the same, irrespective of vaccination status.

There was no confounding effect of any of the variables, and no effect of Season in any of the models. Each one of the final models explained 21–30% of the variation in the outcome variable.

## Discussion

### Effect of Baseline and Initiation of Vaccination

The objective of this study was to determine the effect of initiating vaccination against MYC, PCV2, APP, PRRS, and LAW on the Change in ADD for weaner and finishing pigs at herd level. We found that the Baseline ADD level had a persistent impact, being significant in all models and with more or less the same degree of impact on the Change in ADD for weaners as for finishing pigs. The effect of Baseline ADD in the models showed that, the higher the Baseline ADD in a herd, the larger the decrease in ADD seen over time. This was a general effect, regardless of vaccination, as the effect did not differ between vaccinating and non-vaccinating herds.

### Change in Antimicrobial Use in Danish Pig Herds

Overall, we found an average change in antimicrobial use around 0, for both weaner and finishing pigs, regardless of vaccination status. Some herds experienced a decrease in ADD and, in general, these herds had a high Baseline ADD. Other herds remained at a more or less constant level of ADD, or even increased in ADD over time. The latter were herds with an average or low Baseline ADD. Therefore, it seems reasonable that the Baseline ADD is an important variable to include, when looking at Change in ADD in individual pig herds. Still, the level of Baseline ADD and the level of Change in ADD in a herd are influenced by many other factors. As we see from the final models in this study, these two variables, although important, only explained between 1/4 and 1/3 of the total variation of the observed Change in ADD in the herds included. This illustrates that Change in ADD in pig herds is a multifactorial and very complex measure.

One important factor to determine the Change in ADD must be disease occurrence. Producers experiencing disease problems will, in collaboration with the veterinarian, put in place measures to reduce disease and its consequences. This could result in a decrease in antimicrobial use over time. For vaccinating herds, the decrease observed in the present study was most likely due to the effect of vaccination. For non-vaccinating herds, other measures may have been used, such as type of feed, internal biosecurity (including ways of immunizing sows), and way of purchasing breeding animals.

Herds with no disease problems are at a constant risk of getting disease outbreaks. This is either due to the presence or introduction of various infectious disease agents, which can result in a sudden increase in antimicrobial use. The estimated change in antimicrobial use in pig herds over time, regardless of vaccination status, showed signs of regression toward the mean. This makes sense as, in population-based distributions, the conditional expectation of values located in the tails are typically closer to the overall mean, than to its observed value. Therefore, herds with high consumption are more likely to present a decrease, and herds with low consumption are more likely to increase.

An increase in ADD for vaccinating herds should not be interpreted as a missing effect of the vaccines. The increase may be a result of the increased occurrence of diseases other than the one being vaccinated against. In this study, we used the total ADD, since the validity of the different disease indications varies, and some prescriptions do not have an indication assigned to it (VetStat data, unpublished). They would, therefore, not be included, if the ADDs were split into disease indications.

### Effect of Restrictions from Authorities

A reason for reducing antimicrobial use, other than mitigating disease in a pig herd, could be demands from authorities. In general, there has been much focus on reducing antimicrobial use in the general pig population in Denmark, as also seen in many other EU countries. Demands from Danish authorities increased after 2010, when the Yellow Card Scheme was implemented, and an effect on the antimicrobial level was seen already from mid-2010, when permitted limits were announced (2). This effect can also be confirmed by the data included in this study, showing that the Change in ADD was affected by year (before and after 2010), especially in interaction with ADD Baseline. In the same period when the antimicrobial use decreased in Danish pig herds, an increased purchase of vaccines was observed, especially against MYC, PCV2, and APP. This probably reflects that some swine producers increased their use of vaccines, as an alternative to antimicrobial treatment.

There was no significant effect of interactions between initiation of vaccination and year in any of the models, indicating that the decrease in ADD seen after 2010 was not affected by initiation of vaccination. Still, some herds probably succeeded in reducing their antimicrobial consumption followed by initiation of vaccination (2). Other producers possibly adjusted their herd management, quality of feed, or level of biosecurity, in order to comply with the new restrictions. Some farmers probably also reduced their antimicrobial use of solely psychological reasons, by reducing their probability of getting a Yellow Card, if they were close to the Yellow Card limits. This kind of impact is difficult to determine and is out of the scope of this study.

### Effect of Combination of Vaccines

It is generally believed that preventing several diseases through vaccination can have a larger effect, than the effect of preventing the sum of each of them. In this study, there was a significant effect of the interaction between initiating vaccination in combination with existing vaccine programs. This effect on ADD was observed for initiation of vaccination against LAW with concurrent use of PRRS vaccines in finishers (Figure 3). It was not expected that using two vaccines in combination would result in an increased Change in ADD, implying an increased use of ADD. This probably reflects that there was clinical disease due to these agents prior to the initiation of vaccination compared to the herds not vaccinating, resulting in an apparent missing effect of the vaccines due to reverse causality.

### Explanations for Lack of Effect of Vaccines

The lack of significant effect of vaccines on the antimicrobial use should not be interpreted as an indication of the vaccines not being effective. This study paid attention to the effect of initiation of vaccination, but it did not include long-term effects. Moreover, herds included in this study could have initiated vaccination for various reasons, which were unknown at the time of the study. Vaccines should prevent disease in individual animals but can also be used as a control measure at herd level.

Register data—as used here—include both herds with severe problems related to disease and herds in which vaccination is used as a preventive measure or required by the buyer. Producers who export 7 or 30 kg pigs to other countries sell their pigs for a higher price, if the pigs are vaccinated according to the requirements of the purchasing farmer. For farmers in Germany, this scenario is applicable, since it is a requirement that the pigs are vaccinated against PCV2. Exporting of pigs was included as a variable in each model but was only significant in the model testing the effect of initiating vaccination against PCV2 on the antimicrobial use in weaned pigs. It was shown that herds exporting weaned pigs had a larger increase in consumption of antimicrobials than herds which do not. No significant effect of the interaction between Export and Vaccination was observed, so the effect was general for both vaccinating and non-vaccinating herds.

Vaccinating herds were compared to a group of herds, which did not use the vaccines in question during the study period. The reason for not using that vaccine could be that there was no need for it, meaning that non-vaccinating herds could be herds with better animal health, and therefore, it would be more difficult to obtain an effect of vaccination. In line with this, the antimicrobial use was likely a measure of disease, to some extent. However, high use in a Danish context (above 28 ADD per 100 weaners per day, and six ADD per 100 finishers per day) is not necessarily equal to substantial disease problems and poor pig health. Therefore, a positive effect of vaccination may be more difficult to see in the country, with a more visible increase in antimicrobial use, since the Baseline is already at a low level, when compared to many other EU countries (26). In the study by Raith et al. (14), a significant decline in the use of antimicrobials was found after initiation of the PCV2 vaccine program in Austrian pig herds. However, herds in that study reduced their antimicrobial use to a level that would be considered high, in a Danish context (0.56 ADDkg/kg/year, corresponding to 7.7 ADD/100 animals per day, for finishing pigs). Again, this illustrates the impact of the Baseline ADD, which should always be taken into account, when looking at Change in ADD and the effect of vaccination on antimicrobial use.

Temtem et al. (10) found the same lack of effect, or even apparent reverse causality, between vaccination and antimicrobial use. Even though we have taken more information into account, resulting in models with a higher explanatory degree, there is still some variance that cannot be explained with the available variables and models. This variance could reflect some psychologically based reasons, which are not directly measurable. Using vaccines in a herd may represent a different mindset of the producer or the responsible veterinarian, compared to herds not using vaccines. Producers using vaccines may have a general higher perception of the necessity to control and prevent diseases. For a producer, the use of vaccines and antimicrobials, probably in combination, might be the best way of having a successful production with fewer and less sick animals. This could also be influenced by some veterinarians being more likely to suggest the use of vaccines than others.

### Data Availability

Denmark is a country with a large pig production. Only a proportion of Danish pig herds were included in this study, due to different reasons and criteria. Some herds initiated vaccination before the beginning of the study period in 2007 and were, therefore, not included in the study. Only herd types allowing us to follow the pigs from vaccination in the sow herd, through weaning and finishing, were selected for this study. Other types of herds, for example herds with only one age group registered, also vaccinated against the five agents of interest within the study period. But for these types of herds, it would have been impractical to trace back and forth the vaccine dosages and antimicrobial prescriptions to include. The sample of herds included here covered around 50–70% of all Danish vaccinating herds, depending on the study (VetStat data, unpublished).

Some herds were excluded due to extreme or negative values. These could have been further investigated, but that would require a large amount of time-consuming manual work, to identify and correct the reason for these outliers. Only a few percent of the total number of herds were excluded in each study, due to extreme or negative values. Hence, this should not have biased the results.

In the present study, it was necessary to loosen up on the criterion regarding number of vaccine doses purchased. Not many herds would have been included in the study testing effect of APP, PRRS, and LAW, if the same criteria were used as for the study testing effect of MYC and PCV2. This may reflect that these vaccines are applied differently in Danish pig herds. This could, for example, reflect the use of vaccines for other age groups than what they are licensed for. Another explanation could be the use of half dosages, or not using the vaccine continuously throughout the year. We knew that herds included in the study had purchased vaccines for at least 1 year, but it was difficult to get more information than that, besides the number of doses purchased and the number of animals expected to be vaccinated. Again, this could also explain the missing effect of initiating vaccination against these agents.

## Conclusion

This study provided little support for the hypothesis that vaccination against five common endemic diseases provides a plausible strategy to reduce antimicrobial use in Danish pig herds, overall speaking. Still, vaccination can be an asset in some situations.

## Author Contributions

AK contributed to the design and interpretation of the work, performed the data analyses, and drafted the manuscript. LK contributed by re-evaluating and re-running the analyses for the models containing interactions between vaccines, producing the plots and figures, and collaborated in the interpretation and new text for the revised version. LN and LA contributed to the design and interpretation of the work and revised the manuscript. All the authors did the final approval of the version to be published and hereby agreed to be accountable for all aspects of the work.

## Conflict of Interest Statement

The authors declare that the research was conducted in the absence of any commercial or financial relationships that could be construed as a potential conflict of interest.
