# Supplementary material for: No Clear Effect of Initiating Vaccination against Common Endemic Infections on the Amounts of Prescribed Antimicrobials for Danish Weaner and Finishing Pigs during 2007–2013
Source: Front Vet Sci. 2017 Jan 16;3:120. doi: 10.3389/fvets.2016.00120 (PMC5237653; doi:10.3389/fvets.2016.00120)
Supplement: Supplementary file 1 [file Table_1.pdf]

Supplementary Material

No Clear Effect of Initiating Vaccination Against Common Endemic Infections on the Amounts of Prescribed Antimicrobials for Danish Weaner and Finishing Pigs during 2007-2013

Amanda Brinch Kruse*, Leonardo Víctor de Knegt, Liza Rosenbaum Nielsen and Lis Alban

*** Correspondence:** Corresponding Author: amanda@sund.ku.dk

Supplementary Tables 1 to 16

Supplementary Table 1. Descriptive statistics of mean change in ADD/100 weaners/day, baseline ADD/100 finishers/day and number of sows stratified by categorical variables including number of observations in each group for the study testing effect of initiation of vaccination against *M. hyopneumoniae* on the change in ADD/100 weaners/day.

	**Herds with initiation of vaccination N(source herds)=110**				**Herds with no vaccination N(source herds)=172**			
	**N**	**Change in ADD**	**Baseline ADD**	**Sows**	**N**	**Change in ADD**	**Baseline ADD**	**Sows**
**Total**	**110**	**0.7**	**12.8**	**432**	**172**	**1.5**	**10.8**	**375**
**Year**								
<2010	43	3.1	12.4	364	99	2.2	10.8	358
≥2010	67	-0.9	13.2	476	73	0.5	10.9	397
**Quarter**								
1	25	0.5	13.3	418	46	1.1	11.4	382
2	22	1.6	13.0	390	40	2.0	11.4	324
3	21	0.6	13.3	459	38	0.7	11.7	391
4	42	0.4	12.3	450	48	2.0	9.1	398
**SPF**								
SPF	90	1.3	12.4	473	142	1.4	11.0	386
Non-SPF	20	-2.0	15.0	250	30	1.7	9.9	323
**Export**								
Yes	4	9.9	11.9	738	22	0.1	13.4	451
No	106	0.4	12.9	421	150	1.7	10.4	364
**PCV2-vac**								
Yes	63	-0.1	13.6	467	12	3.6	10.7	430
No	47	1.8	11.8	386	160	1.3	10.8	371
**APP-vac**								
Yes	31	-0.8	14.9	444	13	6.8	9.0	436
No	79	1.3	12.0	428	159	1.1	11.0	370
**PRRS-vac**								
Yes	26	-1.4	14.9	527	16	3.8	12.9	412
No	84	1.3	12.2	403	156	1.2	10.6	371
**LAW-vac**								
Yes	10	0.7	12.9	441	8	2.6	10.3	441
No	100	0.7	12.8	431	164	1.4	10.8	372

Supplementary Table 2. Descriptive statistics of mean change in ADD/100 finishers/day, baseline ADD/100 finishers/day and number of sows stratified by categorical variables including number of observations in each group for the study testing effect of initiation of vaccination against *M. hyopneumoniae* on the change in ADD/100 finishers/day.

	**Herds with initiation of vaccination N(source herds)=103 N(receiving herds)=138**				**Herd with no vaccination N(source herds)=134 N(receiving herds)=164**			
	**N**	**Change in ADD**	**Baseline ADD**	**Sows**	**N**	**Change in ADD**	**Baseline ADD**	**Sows**
**Total**	**138**	**-0.3**	**2.9**	**482**	**164**	**-0.1**	**2.6**	**377**
**Year**								
<2010	57	-0.1	2.6	412	69	-0.1	2.7	338
≥2010	81	-0.4	3.1	532	95	-0.1	2.6	405
**Quarter**								
1	39	-0.1	3.0	453	56	-0.1	3.0	377
2	26	-0.2	2.7	410	42	-0.1	2.2	401
3	26	-0.3	2.9	533	35	-0.2	3.1	360
4	47	-0.6	3.0	519	31	-0.2	2.0	364
**SPF**								
SPF	118	-0.3	3.0	522	135	0.0	2.6	393
Non-SPF	20	-0.2	2.2	252	29	-0.7	2.6	303
**Export**								
Yes	61	-0.5	3.2	513	85	-0.1	2.6	436
No	77	-0.2	2.7	458	79	-0.2	2.6	313
**PCV2-vac**								
Yes	79	-0.5	3.3	516	8	-0.4	1.8	288
No	59	0.0	2.4	437	156	-0.1	2.7	382
**APP-vac**								
Yes	33	-0.9	3.7	538	14	-0.3	2.6	473
No	105	-0.1	2.7	465	150	-0.1	2.6	368
**PRRS-vac**								
Yes	32	-0.6	3.8	619	10	-1.0	2.3	426
No	106	0.2	2.7	441	154	-0.1	2.6	374
**LAW-vac**								
Yes	13	0.0	3.6	765	8	-0.5	2.1	445
No	125	-0.3	2.8	453	156	-0.1	2.6	374

Supplementary Table 3. Descriptive statistics of mean change in ADD/100 weaners/day, baseline ADD/100 weaners/day and number of sows stratified by categorical variables including number of observations in each group for the study testing effect of initiation of vaccination against PCV2 on the change in ADD/100 weaners/day.

	Herds with initiation of vaccination N(source herds)=334				Herds with no vaccination N(source herds)=149			
	**N**	**Change in ADD**	**Baseline ADD**	**Sows**	**N**	**Change in ADD**	**Baseline ADD**	**Sows**
**Total**	**334**	**0.0**	**13.2**	**421**	**149**	**1.1**	**11.0**	**321**
**Year**								
<2010	189	0.4	14.0	417	96	2.0	10.4	303
≥2010	145	-0.5	12.2	425	53	-0.5	12.1	354
**Quarter**								
1	95	-0.2	12.2	430	45	1.9	10.9	310
2	95	0.2	13.2	431	35	-0.9	11.3	302
3	74	-0.1	13.6	409	37	1.2	12.0	319
4	70	0.1	14.0	406	32	1.9	9.6	361
**SPF**								
SPF	243	-0.2	13.0	444	102	1.6	10.9	353
Non-SPF	91	0.5	13.6	360	47	0.0	11.1	252
**Export**								
Yes	181	0.4	13.7	476	64	2.6	10.5	399
No	153	-0.5	12.6	355	85	-0.1	11.3	262
**MYC-vac**								
Yes	224	0.5	13.5	428	50	1.9	11.0	283
No	110	-0.9	12.6	405	99	0.7	10.9	340
**APP-vac**								
Yes	91	0.4	13.8	417	23	1.9	12.4	371
No	243	-0.1	13.0	422	126	0.9	10.7	312
**PRRS-vac**								
Yes	90	0.5	13.2	430	11	1.2	11.6	282
No	244	-0.2	13.2	417	138	1.1	10.9	324
**LAW-vac**								
Yes	23	-2.1	14.5	505	3	-1.5	7.7	212
No	311	0.2	13.1	414	146	1.1	11.0	323

Supplementary Table 4. Descriptive statistics of mean change in ADD/100 finishers/day, baseline ADD/100 finishers/day and number of sows stratified by categorical variables including number of observations in each group for the study testing effect of initiation of vaccination against PCV2 on the change in ADD/100 finishers/day.

	**Herds with initiation of vaccination N(source herds)=278 N(receiving herds)=365**				**Herds with no vaccination N(source herds)=130 N(receiving herds)=158**			
	**N**	**Change in ADD**	**Baseline ADD**	**Sows**	**N**	**Change in ADD**	**Baseline ADD**	**Sows**
**Total**	**365**	**0.0**	**3.0**	**437**	**158**	**0.1**	**2.5**	**331**
**Year**								
<2010	214	0.2	3.0	454	81	0.3	2.4	290
≥2010	151	-0.3	2.9	414	77	-0.2	2.5	374
**Quarter**								
1	96	-0.1	3.1	467	50	0.2	2.3	304
2	122	0.1	2.8	446	37	-0.6	2.5	374
3	79	0.0	3.2	418	36	0.6	2.7	326
4	68	-0.1	2.9	403	35	0.0	2.4	330
**SPF**								
SPF	272	0.0	2.9	463	102	0.0	2.4	355
Non-SPF	93	-0.1	3.2	362	56	0.3	2.6	288
**Export**								
Yes	199	0.1	3.0	497	65	0.0	2.8	401
No	166	-0.1	2.9	366	93	0.1	2.2	282
**MYC-vac**								
Yes	240	0.0	3.0	447	57	0.2	2.9	298
No	125	0.0	2.9	419	101	0.0	2.2	350
**APP-vac**								
Yes	93	0.1	3.6	412	40	0.0	2.8	382
No	272	0.0	2.8	446	118	0.1	2.3	314
**PRRS-vac**	84	0.1	3.2	444	9	-0.4	3.5	350
Yes	281	0.0	2.9	435	149	0.1	2.4	330
No								
**LAW-vac**								
Yes	35	0.2	2.8	574	3	0.4	2.3	227
No	330	0.0	3.0	423	155	0.1	2.5	333

Supplementary Table 5. Descriptive statistics of mean change in ADD/100 weaners/day, baseline ADD/100 weaners/day and number of sows stratified by categorical variables including number of observations in each group for the study testing effect of initiation of vaccination against *A. pleuropneumonia* on the change in ADD/100 weaners/day.

	**Herds with initiation of vaccination N(source herds)=105**				**Herds with no vaccination N(source herds)=465**			
	**N**	**Change in ADD**	**Baseline ADD**	**Sows**	**N**	**Change in ADD**	**Baseline ADD**	**Sows**
**Total**	**105**	**-0.2**	**13.6**	**466**	**465**	**0.7**	**11.3**	**410**
**Year**								
<2010	51	0.1	15.3	444	239	1.3	11.2	363
≥2010	54	-0.5	12.1	486	226	0.1	11.4	461
**Quarter**								
1	34	-0.6	13.8	449	127	0.9	10.6	386
2	27	0.6	12.9	461	120	0.2	11.8	410
3	22	1.2	14.0	574	116	0.7	12.5	417
4	22	-1.8	14.0	388	102	1.2	10.4	435
**SPF**								
SPF	68	0.0	14.1	510	382	0.9	11.4	440
Non-SPF	37	-0.5	12.8	383	83	-0.1	10.8	274
**Export**								
Yes	59	0.3	13.8	514	245	0.8	11.3	475
No	46	-0.8	13.4	404	220	0.7	11.4	339
**MYC-vac**								
Yes	83	-0.6	14	472	192	0.9	11.3	420
No	22	1.6	12.4	439	273	0.7	11.3	404
**PCV2-vac**								
Yes	68	-0.6	13.8	496	25	0.8	9.6	470
No	37	0.5	13.3	409	440	0.7	11.4	407
**PRRS-vac**								
Yes	44	-0.3	13.8	561	48	1.0	12.8	410
No	61	-0.1	13.5	396	417	0.7	11.2	411
**LAW-vac**								
Yes	8	-7.2	18.9	355	10	1.2	9.2	396
No	97	0.4	13.2	475	455	0.7	11.4	411

Supplementary Table 6. Descriptive statistics of mean change in ADD/100 finishers/day, baseline ADD/100 finishers/day and number of sows stratified by categorical variables including number of observations in each group for the study testing effect of initiation of vaccination against *A. pleuropneumonia* on the change in ADD/100 finishers/day.

	**Herds with initiation of vaccination N(source herds)=69 N(receiving herds)=89**				**Herds with no vaccination N(source herds)=390 N(receiving herds)=480**			
	**N**	**Change in ADD**	**Baseline ADD**	**Sows**	**N**	**Change in ADD**	**Baseline ADD**	**Sows**
**Total**	**89**	**0.2**	**3.1**	**475**	**489**	**0.1**	**2.6**	**413**
**Year**								
<2010	47	0.4	2.7	447	221	0.1	2.6	369
≥2010	42	-0.1	3.4	508	268	0.0	2.6	449
**Quarter**								
1	30	0.3	3.2	490	150	0.0	2.9	398
2	21	-0.2	3.6	454	125	0.2	2.5	421
3	19	0.8	2.5	495	113	0.0	2.6	408
4	19	-03	2.9	456	101	0.2	2.5	431
**SPF**								
SPF	53	0.3	3.2	532	406	0.1	2.7	444
Non-SPF	36	0.0	2.9	392	83	0.0	2.4	261
**Export**								
Yes	42	0.2	3.3	558	249	0.1	2.7	472
No	47	0.1	2.9	402	240	0.0	2.6	351
**MYC-vac**								
Yes	73	0.2	3.1	471	197	0.0	2.8	426
No	16	0.0	2.9	484	292	0.1	2.5	404
**PCV2-vac**								
Yes	57	0.0	3.4	507	22	0.4	2.1	468
No	32	0.4	2.5	419	467	0.1	2.6	410
**PRRS-vac**								
Yes	36	-0.2	3.5	602	39	-0.2	2.8	425
No	53	0.4	2.8	390	450	0.1	2.6	412
**LAW-vac**								
Yes	5	0.0	3.2	260	9	-0.3	3.3	389
No	84	0.2	3.1	488	480	0.1	2.6	413

Supplementary Table 7. Descriptive statistics of mean change in ADD/100 weaners/day, baseline ADD/100 weaners/day and number of sows stratified by categorical variables including number of observations in each group for the study testing effect of initiation of vaccination against PRRS on the change in ADD/100 weaners/day.

	Herds with initiation of vaccination N(source herds)=190				Herds with no vaccination N(source herds)=456			
	N	Change in ADD	Baseline ADD	Sows	N	Change in ADD	Baseline ADD	Sows
**Total**	**190**	**0.3**	**13.3**	**510**	**456**	**1.5**	**11.2**	**392**
**Year**								
<2010	78	0.9	13.7	513	244	2.2	11.5	356
≥2010	112	-0.1	13.1	508	212	0.7	10.9	433
**Quarter**								
1	54	0.1	13.6	475	117	2.1	10.6	362
2	45	1.1	14.8	577	113	0.6	11.4	386
3	40	-1.7	11.6	474	115	0.8	12.2	385
4	51	1.4	13.1	517	111	2.6	10.7	436
**SPF**								
SPF	134	0.5	13	527	336	1.5	11.2	433
Non-SPF	56	-0.4	14.1	471	120	1.6	11.3	276
**Export**								
Yes	117	0.9	13.3	573	221	1.4	11.5	459
No	73	-0.7	13.4	409	235	1.6	10.9	328
**MYC-vac**								
Yes	138	0.3	13.9	519	197	1.3	11.8	394
No	52	0.3	11.8	488	259	1.6	10.8	390
**PCV2-vac**								
Yes	126	-0.1	13.5	524	19	0.9	9.1	418
No	64	1.0	13.0	483	437	1.5	11.3	390
**APP-vac**								
Yes	61	0.1	14.5	543	73	3.2	12.0	421
No	129	0.4	12.8	495	383	1.2	11.1	386
**LAW-vac**								
Yes	12	-3.1	15.7	610	9	2.9	8.4	364
No	178	0.5	13.2	504	447	1.5	11.3	392

Supplementary Table 8. Descriptive statistics of mean change in ADD/100 finishers/day, baseline ADD/100 finishers/day and number of sows stratified by categorical variables including number of observations in each group for the study testing effect of initiation of vaccination against PRRS on the change in ADD/100 finishers/day.

	**Herds with initiation of vaccination N(source herds)=139 N(receiving herds)=200**				**Herds with no vaccination N(source herds)=434 N(receiving herds)=543**			
	**N**	**Change in ADD**	**Baseline ADD**	**Sows**	**N**	**Change in ADD**	**Baseline ADD**	**Sows**
**Total**	**200**	**-0.1**	**2.8**	**507**	**543**	**0.1**	**2.6**	**406**
**Year**								
<2010	87	0.4	2.4	525	237	0.2	2.5	353
≥2010	113	-0.4	3.1	493	306	0.0	2.7	447
**Quarter**								
1	54	0.2	2.6	457	165	0.1	2.7	388
2	44	0.1	2.8	588	128	0.1	2.5	413
3	45	-0.8	2.8	504	119	-0.1	2.6	393
4	57	0.2	3.0	494	131	0.2	2.6	435
**SPF**								
SPF	133	0.0	2.8	529	412	0.0	2.7	445
Non-SPF	67	-0.1	2.9	464	131	0.3	2.5	285
**Export**								
Yes	122	0.0	2.9	566	271	0.2	2.6	478
No	78	-0.1	2.7	414	272	0.0	2.6	335
**MYC-vac**								
Yes	161	0.1	2.7	507	233	0.1	2.8	423
No	39	-0.6	3.3	509	310	0.1	2.5	394
**PCV2-vac**								
Yes	144	0.0	3.0	534	16	-0.4	2.9	442
No	56	-0.2	2.4	436	527	0.1	2.6	405
**APP-vac**								
Yes	76	0.0	2.8	527	96	0.0	2.7	444
No	124	-0.1	2.8	495	447	0.1	2.6	398
**LAW-vac**								
Yes	18	0.0	2.9	485	10	-0.1	3.6	439
No	182	-0.1	2.8	509	533	0.1	2.6	406

Supplementary Table 9. Descriptive statistics of mean change in ADD/100 weaners/day, baseline ADD/100 weaners/day and number of sows stratified by categorical variables including number of observations in each group for the study testing effect of initiation of vaccination against *L. intracellularis* on the change in ADD/100 weaners/day.

	Herds with initiation of vaccination N(source herds)=81				Herds with no vaccination N(source herds)=570			
	**N**	**Change in ADD**	**Baseline ADD**	**Sows**	**N**	**Change in ADD**	**Baseline ADD**	**Sows**
**Total**	**81**	**0.1**	**11.9**	**515**	**570**	**1.0**	**11.9**	**419**
**Year**								
<2010	23	1.6	13.0	528	307	2.1	11.7	384
≥2010	58	-0.4	11.4	510	63	-0.2	12.1	459
**Quarter**								
1	18	-1.2	12.4	455	162	1.4	10.9	406
2	30	-0.4	11.8	525	144	0.2	12.8	421
3	14	2.5	9.2	543	136	1.0	12.1	399
4	19	0.6	13.4	537	128	1.6	11.7	454
**SPF**								
SPF	71	0.0	11.6	531	388	1.1	11.7	456
Non-SPF	10	1.1	13.6	403	182	0.9	12.1	340
**Export**								
Yes	45	1.5	11.5	602	289	1.2	11.8	499
No	36	-1.6	12.4	406	281	0.9	11.9	336
**MYC-vac**								
Yes	47	-0.4	12.3	537	284	1.1	12.4	430
No	34	0.8	11.2	486	286	1.0	11.3	407
**PCV2-vac**								
Yes	51	-0.1	11.3	546	31	0.2	11.4	492
No	30	0.6	12.9	463	539	1.1	11.9	414
**APP-vac**								
Yes	13	0.1	13.6	491	116	1.6	13.0	461
No	68	0.1	11.6	520	454	0.9	11.6	408
**PRRS-vac**								
Yes	10	0.1	13.7	728	65	0.0	13.8	452
No	71	0.1	11.6	485	505	1.2	11.6	414

Supplementary Table 10. Descriptive statistics of mean change in ADD/100 finishers/day, baseline ADD/100 finishers/day and number of sows stratified by categorical variables including number of observations in each group for the study testing effect of initiation of vaccination against *L. intracellularis* on the change in ADD/100 finishers/day.

	**Herds with initiation of vaccination N(source herds)=71 N(receiving herds)=105**				**Herds with no vaccination N(source herds)=522 N(receiving herds)=662**			
	**N**	**Change in AD**	**Baseline ADD**	**Sows**	**N**	**Change in ADD**	**Baseline ADD**	**Sows**
**Total**	**105**	**0.0**	**3.0**	**571**	**662**	**0.1**	**2.7**	**442**
**Year**								
<2010	28	0.4	2.8	629	309	0.2	2.6	405
≥2010	77	-0.2	3.0	552	353	0.0	2.7	474
**Quarter**								
1	23	-0.1	2.1	508	192	0.0	2.8	419
2	44	0.4	3.1	586	162	0.0	2.6	438
3	16	0.0	2.5	688	160	0.0	2.8	444
4	22	-0.7	4.1	527	148	0.3	2.4	472
**SPF**								
SPF	93	0.0	3.0	580	467	0.0	2.8	481
Non-SPF	12	0.1	2.6	511	195	0.1	2.4	348
**Export**								
Yes	62	0.2	3.3	660	357	0.1	2.7	507
No	43	-0.3	2.6	446	305	0.0	2.6	365
**MYC-vac**								
Yes	55	0.0	2.8	577	319	0.0	2.8	459
No	50	-0.1	3.2	567	343	0.1	2.6	426
**PCV2-vac**								
Yes	70	-0.1	3.3	610	21	0.6	2.7	497
No	35	0.0	2.4	497	641	0.1	2.7	440
**APP-vac**								
Yes	19	1.0	2.9	663	149	-0.1	2.9	459
No	86	-0.2	3.0	552	513	0.1	2.6	437
**PRRS-vac**								
Yes	18	0.9	3.8	846	62	-0.3	3.0	479
No	87	-0.2	2.8	516	600	0.1	2.6	438

Supplementary Table 11. Descriptive statistics of continuous variables in each of the five vaccine studies testing effect of initiation of vaccination against MYC, PCV2, APP, PRRS and LAW on the Change in ADD/100 animals/day for weaners and finishers, respectively.

Variable	Age group	Vaccine study	Min	Q1	Median	Mean	Q3	Max
Change in ADD	Weaners	MYC	-22.7	-2.8	0.9	1.2	4.6	33.8
		PCV2	-27.9	-3.1	0.4	0.3	3.9	30.8
		APP	-30.7	-2.8	0.4	0.6	4.3	37.8
		PRRS	-26.8	-2.6	0.7	1.2	4.5	33.8
		LAW	-27.9	-3.0	0.6	0.9	4.5	33.8
	Finishers	MYC	-7.6	-0.9	-0.1	-0.2	0.6	5.1
		PCV2	-8.8	-0.9	0.1	0.0	0.9	8.3
		APP	-7.6	-0.8	0.1	0.1	1.0	8.3
		PRRS	-7.3	-0.8	0.0	0.1	0.9	10.0
		LAW	-7.3	-0.9	0.0	0.1	1.0	8.3
Baseline ADD	Weaners	MYC	0.2	6.9	10.3	11.6	15.1	39.1
		PCV2	0.2	6.7	11.2	12.5	16.4	39.1
		APP	0.2	6.3	10.3	11.8	15.5	39.1
		PRRS	0.2	6.6	10.4	11.8	15.9	39.2
		LAW	0.0	6.5	10.5	11.9	15.9	39.1
	Finishers	MYC	0.1	1.1	2.1	2.7	3.9	9.9
		PCV2	0.1	1.1	2.3	2.8	4.1	9.9
		APP	0.1	1.1	2.2	2.7	3.7	9.5
		PRRS	0.0	1.2	2.2	2.7	3.7	10.0
		LAW	0.0	1.1	2.2	2.7	3.8	10.0
Sows	Weaners	MYC	100	250	350	397	500	1300
		PCV2	100	250	349	390	487	1500
		APP	100	250	378	421	550	1830
		PRRS	100	250	400	426	556	2080
		LAW	100	250	390	431	550	2080
	Finishers	MYC	105	253	380	425	560	1300
		PCV2	100	250	370	405	530	1500
		APP	100	250	382	422	550	2012
		PRRS	100	260	400	434	580	1690
		LAW	100	265	420	460	600	2012

Supplementary Table 12. Regression outputs of the final linear regression models (weaners) and linear mixed models with random effects of potential confounders (finishers) predicting the change in antimicrobial consumption[a] after initiation of vaccination against *M. hyopneumoniae* (MYC) in selected Danish swine herds between 2007 and 2013.

Age group	Significant variables	Variance	SD	Levels	β	SE	*P*
**Weaners R^2 = 0.25**	Intercept				6.84	1.19	-
	Baseline ADD				-0.64	0.083	<0.001
	Year			<2010	-1.25	1.53	0.42
				≥2010	0	0	
	Baseline * Year[b]			<2010	0.31	0.11	0.005
				≥2010	0	0	
**Finishers R^2 = 0.27**	**Random effects**						
	Source herd	0.19	0.43				
	Residuals	1.81	1.34				
	**Fixed effects**						
	Intercept				0.67	0.23	-
	Baseline ADD				-0.48	0.038	<0.001
	SPF			Yes	0.58	0.22	0.01
				No	0	0	

[a] Change measured in ADD/100 pigs/day. One ADD (Animal Defined Daily Dose) is defined as “the assumed average maintenance dose per day for the main indication in a specified species” (DANMAP 2009 - Use of antimicrobial agents and occurrence of antimicrobial resistance in bacteria from food animals, food and humans in Denmark. http://www.danmap.org/Downloads/Reports.aspx).

[b] Multiplied variables indicate interaction terms.

Supplementary Table 13. Regression outputs of the final linear regression models (weaners) and linear mixed models with random effects of potential confounders (finishers) predicting the change in antimicrobial consumption[a] after initiation of vaccination against PCV2 in selected Danish swine herds between 2007 and 2013.

Age group	Significant variables	Variance	SD	Levels	β	SE	*P*
**Weaners** R^2 **= 0.27**	Intercept				4.90	0.74	-
	Baseline ADD				-0.52	0.040	<0.001
	Year			<2010	1.79	0.62	0.004
				≥2010	0	0	
	Export			Yes	1.71	0.61	0.005
				No	0	0	
**Finishers** R^2 **= 0.24**	**Random effects**						
	Source herd	0.076	0.28				
	Residuals	3.16	1.78				
	**Fixed effects**						
	Intercept				0.64	0.22	
	Baseline ADD				-0.43	0.037	<0.001
	Sows				0.00077	0.00038	0.05
	Year			<2010	0.52	0.16	0.001
				≥2010	0	0	

[a] Change measured in ADD/100 pigs/day. One ADD (Animal Defined Daily Dose) is defined as "the assumed average maintenance dose per day for the main indication in a specified species" (DANMAP 2009 - Use of antimicrobial agents and occurrence of antimicrobial resistance in bacteria from food animals, food and humans in Denmark. http://www.danmap.org/Downloads/Reports.aspx).

[b] Multiplied variables indicate interaction terms.

Supplementary Table 14. Regression outputs of the final linear regression models (weaners) and linear mixed models with random effects of potential confounders (finishers) predicting the change in antimicrobial consumption[a] after initiation of vaccination against *A. pleuropneumoniae* (APP) in selected Danish swine herds between 2007 and 2013.

Age group	Significant variables	Variance	SD	Levels	β	SE	*P*
	Intercept				5.43	1.014	-
	Baseline ADD				-0.64	0.057	<0.001
	Sows				0.0044	0.0015	0.004
**Weaners**	Year			<2010	0.61	1.40	0.66
**R^2 = 0.26**				≥2010	0	0	
	Sows * Year‡			<2010	-0.0049	0.0024	0.04
	Baseline * Year‡			≥2010	0	0	
				<2010	0.25	0.074	<0.001
				≥2010	0	0	
	**Random effects**						
	Source herd	0.60	0.78				
**Finishers**	Residuals	2.51	1.58				
**R^2 = 0.29**							
	**Fixed effects**						
	Intercept				1.37	0.12	-
	Baseline ADD				-0.48	0.036	<0.001

[a] Change measured in ADD/100 pigs/day. One ADD (Animal Defined Daily Dose) is defined as "the assumed average maintenance dose per day for the main indication in a specified species" (DANMAP 2009 - Use of antimicrobial agents and occurrence of antimicrobial resistance in bacteria from food animals, food and humans in Denmark. http://www.danmap.org/Downloads/Reports.aspx).

[b] Multiplied variables indicate interaction terms.

Supplementary Table 15. Regression outputs of the final linear regression models (weaners) and linear mixed models with random effects of potential confounders (finishers) predicting the change in antimicrobial consumption[a] after initiation of vaccination against PRRS in selected Danish swine herds between 2007 and 2013.

**Age group**	**Significant variables**	**Variance**	**SD**	**Levels**	**β**	**SE**	***P***
**Weaners R^2 = 0.21**	Intercept				5.70	0.59	-
	Baseline ADD				-0.49	0.037	<0.001
	Year			<2010	1.70	0.55	0.002
				≥2010	0	0	
	APP-vac			Yes	1.65	0.68	0.02
				No	0	0	
**Finishers R^2 = 0.24**	**Random effects**						
	Source herd	0.57	0.76				
	Residuals	2.55	1.60				
	**Fixed effects**						
	Intercept				1.37	0.16	-
	Baseline ADD				-0.53	0.046	<0.001
	Year			<2010	0.52	0.16	0.5
				≥2010	0	0	
	Baseline*Year[‡]			<2010	0.14	0.066	0.03
				≥2010	0	0	

[a] Change measured in ADD/100 pigs/day. One ADD (Animal Defined Daily Dose) is defined as "the assumed average maintenance dose per day for the main indication in a specified species" (DANMAP 2009 - Use of antimicrobial agents and occurrence of antimicrobial resistance in bacteria from food animals, food and humans in Denmark. http://www.danmap.org/Downloads/Reports.aspx).

[b] Multiplied variables indicate interaction terms.

Supplementary Table 16. Regression outputs of the final linear regression models (weaners) and linear mixed models with random effects of potential confounders (finishers) predicting the change in antimicrobial consumption[a] after initiation of vaccination against *L. intracellularis* (LAW) in selected Danish swine herds between 2007 and 2013.

Age group	Significant variables	Variance	SD	Levels	β	SE	*P*
	Intercept				5.32	0.90	-
	Baseline ADD				-0.56	0.057	<0.001
**Weaners**	Sows				0.0025	0.0011	0.02
**R^2 = 0.21**	Year			<2010	0.014	1.029	1.0
				≥2010	0	0	
	Baseline * Year[‡]			<2010	0.20	0.074	<0.007
				≥2010	0	0	
	**Random effects**						
	Source herd	0.45	0.67				
	Residuals	2.46	1.57				
	**Fixed effects**						
	Intercept				1.38	0.11	-
**Finishers**	Baseline ADD				-0.48	0.031	<0.001
**R^2 = 0.30**	LAW&PRRS vaccination						0.03
				LAW-PRRS+ vs. LAW-PRRS-	--0.20	0.23	0.81[†]
				LAW+PRRS- vs. LAW-PRRS-	-0.23	0.21	0.67[†]
				LAW+PRRS+ vs. LAW-PRRS-	1.15	0.44	0.04[†]
				LAW+PRRS- vs. LAW-PRRS+	-0.03	0.29	0.10[†]
				LAW+PRRS+ vs. LAW-PRRS+	1.35	0.49	0.03[†]
				LAW+PRRS+ vs. LAW+PRRS-	1.38	0.48	0.02[†]

[a] Change measured in ADD/100 pigs/day. One ADD (Animal Defined Daily Dose) is defined as "the assumed average maintenance dose per day for the main indication in a specified species" (DANMAP 2009 - Use of antimicrobial agents and occurrence of antimicrobial resistance in bacteria from food animals, food and humans in Denmark. http://www.danmap.org/Downloads/Reports.aspx).

[b] Multiplied variables indicate interaction terms.

[†] Tukey's 'Honest Significant Difference' method
